# Sex differences in oxidative stress level and antioxidative enzymes expression and activity in obese pre-diabetic elderly rats treated with metformin or liraglutide

**DOI:** 10.3325/cmj.2021.62.215

**Published:** 2021-06

**Authors:** Anita Matić, Rosemary Vuković, Marija Heffer, Marta Balog, Vedrana Ivić, Robert Gaspar, Eszter Ducza, Kalman F. Szucs, Adrienn Seres, Sandor G. Vari, Ines Drenjančević

**Affiliations:** 1 Department of Physiology and Immunology, Faculty of Medicine, University Josip Juraj Strossmayer Osijek, Osijek, Croatia; 2 Department of Biology, University Josip Juraj Strossmayer Osijek, Osijek, Croatia; 3 Department of Medical Biology and Genetics, Faculty of Medicine Osijek, University Josip Juraj Strossmayer of Osijek, Osijek, Croatia; 4 Department of Pharmacology and Pharmacotherapy, Faculty of Medicine, University of Szeged, Szeged, Hungary; 5 Department of Pharmacodynamics and Biopharmacy, Faculty of Pharmacy, University of Szeged, Szeged, Hungary; 6 International Research and Innovation in Medicine Program, Cedars-Sinai Medical Center, Los Angeles, CA, USA; 7 Regional Cooperation for Health, Science and Technology (RECOOP HST) Association, Debrecen, Hungary

## Abstract

**Aim:**

To determine the effects of metformin or liraglutide on oxidative stress level and antioxidative enzymes gene transcription and activity in the blood and vessels of pre-diabetic obese elderly Sprague-Dawley (SD) rats of both sexes.

**Methods:**

Male and female SD rats were assigned to the following groups: a) control group (fed with standard rodent chow); b) high-fat and high-carbohydrate diet (HSHFD) group fed with HSHFD from 20-65 weeks of age; c) HSHFD+metformin treatment (50 mg/kg/d s.c.); and d) HSHFD+liraglutide treatment (0.3 mg/kg/d s.c). Oxidative stress parameters (ferric reducing ability of plasma and thiobarbituric acid reactive substances) and superoxide dismutase (SOD), catalase (CAT), and glutathione peroxidase (GPx) activity and gene transcription were determined from serum, aortas, and surface brain blood vessels (BBV).

**Results:**

HSHFD increased body weight in both sexes compared with the control group, while liraglutide prevented this increase. Blood glucose level did not change. The liraglutide group had a significantly increased antioxidative capacity compared with the HSHFD group in both sexes. The changes in antioxidative enzymes’ activities in plasma were more pronounced in male groups. The changes in gene expression of antioxidative enzymes were more prominent in microvessels and may be attributed to weight gain prevention.

**Conclusions:**

Obesity and antidiabetic drugs caused sex-related differences in the level of antioxidative parameters. Liraglutide exhibited stronger antioxidative effects than metformin. These results indicate that weight gain due to HSHFD is crucial for developing oxidative stress and for inhibiting antioxidative protective mechanisms.

Lifestyle and diet changes are related to the development of many chronic cardiometabolic diseases, such as obesity, type 2 diabetes mellitus (T2DM), cardiovascular disease (CVD), and atherosclerosis (1-3). Their common denominator is oxidative stress (4), an imbalance between the production of reactive oxygen species (ROS; such as superoxide, hydrogen peroxide, etc) and antioxidative capacity (5). Oxidative stress is caused by different types of chronic or acute dietary protocols, such as high-fat-high-carbohydrate diet (HSHFD) or high dietary intake of saturated fatty acids and trans-fatty acids, via multiple biochemical mechanisms (6-8). The high level of free radicals is decreased through a synergistical action of antioxidant enzymes (superoxide dismutases [SOD], glutathione peroxidases [GPx], catalase [CAT], glutathione S-transferase, thioredoxin reductase, etc) (9). Antioxidant status in blood vessel tissue and blood samples can help us assess the impact of obesity and T2DM on the cardiovascular system. For example, the activity of antioxidative enzymes in obese individuals is lower than that of non-obese individuals, and probably underlies the obesity-related health problems (10). Besides T2DM, obesity is often accompanied by an increased risk of CVD, including coronary artery disease, stroke, and peripheral arterial disease (11). All of these diseases present with endothelial dysfunction due to a reduced bioavailability of vasodilator nitric oxide, inflammation, increased free radicals and cytokines production, and oxidation of low-density lipoproteins (12,13). In addition, obesity and T2DM are often associated with hyperinsulinemia, a condition characterized by a glucose transport disorder, pancreatic β-cell dysfunction, increased levels of oxidative stress, and inflammation (14,15).

Liraglutide, a glucagon-like peptide-1 agonist (GLP-1), decreases blood glucose by potentiating glucose-dependent insulin secretion, by enhancing β-cells growth, and by reducing food intake and body weight (16). One of its effects is also the reduction of the plasma ROS level in T2DM patients (17). Liraglutide decreases oxidative stress in diabetes by the activation of cAMP, epidermal growth factor receptor-PI3K, and protein kinase C pathways, and Nrf-2 activation. These processes increase the antioxidant capacity or antioxidative enzymes expression in tissues, the parameters that are often altered in diabetes (18,19). Animal studies showed liraglutide to improve insulin resistance in the liver and adipose tissue of diabetic mice (20) and to affect vasculature by increasing microvascular recruitment and blood flow (21). Furthermore, liraglutide induced cardioprotection and reduced death rates from cardiovascular causes in T2DM (21).

Metformin mechanisms of action include improving insulin sensitivity and reducing glycemia without significantly increasing hypoglycemia event rate (22). Similar to liraglutide, metformin has antioxidant and anti-inflammatory properties (23). It reduces the expression of NF-kB, a transcription factor involved in inflammation, by inhibiting IL-8 and IL-1α inflammatory cytokines, and inhibits the differentiation of monocytes into macrophages. It contributes to the reduction of oxidative stress by reducing hydrogen peroxide level by activating catalase or by reducing the transcription of NADPH oxidase 4 (23). Furthermore, like liraglutide, metformin has a protective effect on vasculature. For example, it can inhibit various steps of angiogenesis, including endothelial cell proliferation in retinal vascular endothelial cell culture, or reduce spontaneous intraretinal neovascularization (24).

This study, for the first time in the literature, assessed the gene expression of antioxidant enzymes (SOD, GPx, and CAT) in the aortas and surface brain blood vessels of obese animals of both sexes, and explored the effects of liraglutide and metformin treatment on the expression of these genes.

## Materials and methods

### Setting

The sampling was performed at the Department of Pharmacodynamics and Biopharmacy, Faculty of Pharmacy, University of Szeged, Hungary. All molecular measurements were carried out in the Laboratory for Molecular and Clinical Immunology at the Department of Physiology and Immunology, Faculty of Medicine, and at the Laboratory for Biochemistry, Department of Biology, Josip Juraj Strossmayer University of Osijek. The study (including the feeding protocol, drug treatments, sampling, and sample processing to final results) lasted from June 2015 until November 2016. All experimental procedures conformed to the European Communities Council Directives (2010/63/EU) and were approved by the Hungarian National Scientific Ethics Committee on Animal Experimentation (IV/3084/2016).

### Experimental animals

Male and female Sprague-Dawley rats (Charles River, Germany) were given rodent pellet diet and drinking water *ad libitum*. They were housed four rats per cage (polypropylene cages Type IV, floor area 1800 cm^2^) under controlled temperature (20-23 °C) in humidity- (40%-60%) and light- (12 h light/dark regime) regulated rooms. Commercially available carbohydrate- and fat-rich food (56% of carbohydrates and 12% of crude fat) was purchased from Altromin Spezialfutter GmbH & Co (Lage, Germany).

### Studied groups

The animals of both sexes were randomized into four groups. A total of 31 female and 32 male rats were included in the study. However, some animals did not survive until the end of the protocol, so the final number of animals was 28 female and 29 male rats. The groups were as follows:

a) control group (initial: n_female_ = 7; n_male_ = 8, final: n_female_ = 6; n_male_ = 7) – animals were fed with standard rat chow during the whole protocol;

b) HSHFD group (initial: n_female_ = 8; n_male_ = 8, final: n_female_ = 8; n_male_ = 6) – carbohydrate- and fat-rich diet for 20 weeks, from the 45th week to 65th week of age;

c) HSHFD+metformin (initial: n_female_ = 8; n_male_ = 8, final: n_female_ = 7; n_male_ = 8) – carbohydrate- and fat-rich diet from the 45th week of age + metformin treatment (50 mg/kg/d s.c.) from the 51st-65th week of age; and

d) HSHFD+liraglutide (initial: n_female_ = 8; n_male_ = 8, final: n_female_ = 7; n_male_ = 8) – carbohydrate- and fat-rich diet from the 45th week of age + liraglutide treatment (0.3 mg/kg/d s. c.) from the 51st-65th week of age.

Metformin (Sigma Aldrich, Budapest, Hungary) and liraglutide (Creative Peptides INC, New York, NY, USA) were administered each morning between 9.00 and 10.00 am. The drugs were dissolved in a special buffer containing 0.5 mg disodium hydrogen phosphate dihydrate, 4.7 mg propylene glycol, and 1.8 mg phenol in 1-mL water solution (pH 8.5). The control group was treated with 0.1 mL buffer each day of treatment.

### Sampling

The baseline body weight results represent the body weight of 45-week-old rats that received no treatment or HSHFD diet and were measured before sacrifice. Blood glucose concentration was also measured before sacrifice at 8 am with OneTouch® UltraMini® Glucose Meter (Milpitas, CA, USA), after a 16-hour fast. Blood was collected from the tail vein of awake, non-anesthetized rats that were put in a restrainer. At the end of the protocol, animals were anesthetized with isoflurane (Forane® isofluranum, Abbott Laboratories Ltd, Queenborough, UK). Blood (serum and plasma) samples were collected for enzyme activity and oxidative stress measurements, while BBV and aortas were collected for gene expression measurements. Surface BBV were isolated with forceps and dissection microscope and quick-frozen in liquid nitrogen. Thoracic aortas were stored in the same fashion.

### mRNA expression studies

Samples and total RNA were homogenized with One Step RNA Reagent (Bio Basic Inc, Markham, Canada) according to the manufacturer`s protocol. RNA was purified from gDNA with Deoxyribonuclease I kit (Sigma-Aldrich, St. Louis, MO; USA), and the obtained cDNA was synthesized with High Capacity cDNA kit (Applied Biosystems, Waltham, MA, USA). RNA expression was determined with SsoFast EvaGreen Supermix (Bio Rad, Hercules, CA, USA). The following genes: *Sod* isoforms (Cu/Zn *Sod*, *MnSod*, *EC-Sod*), *Gpx1* and *Gpx4*, and *Cat* were normalized to the expression of the housekeeping genes hypoxanthine-guanine phosphoribosyltransferase (*Hprt*) and 18s. The results obtained with *Hprt* were more consistent, so this gene was used in further analysis. The gene expression analysis was performed with BioRad CFX96 (25,26).

### Antioxidant enzyme activities

Antioxidant enzyme activities were measured from plasma samples. All measurements were performed as previously described (25,27-29). The enzyme activities were expressedas units per milligram protein. Enzyme activity assay was performed with a Lambda 25UV-Vis spectrophotometer equipped with a UV WinLab 6.0 software package (PerkinElmer For the Better, Waltham, MA, USA). The protein concentration in the samples (mg/mL) was determined with Bradford reagent at 595 nm. Bovine serum albumin was used as a standard.

### Oxidative stress markers

Serum levels of an oxidative stress marker (thiobarbituric acid reactive substances; TBARS) and plasma antioxidant capacity (ferric reducing ability of plasma; FRAP) were measured spectrophotometrically. TBARS was used for measuring the oxidative stress level, more precisely the products of lipid peroxidation with malondialdehyde as standard (μM/L MDA) at 572 nm and 532 nm. FRAP (mM/L Trolox) is a spectrophotometric method used to determine the total amount of antioxidants in the sample, ie, their ability to reduce Fe^3+^ ion into the Fe^2+^ ion. Both methods have been described in detail previously (25-30). The values were obtained by Nanophotometer P300 UV (Implen GmbH, Schatzbogen, Germany).

### Statistical analysis

The sample size was determined with the Sigma Plot v 11.0 program (Systat Software, Inc. San Jose, CA, USA). To obtain the power of 0.8, p value less than 0.05, and the minimum expected difference of 0.25, at least 4 animals per group were required. The normality of distribution was assessed with the Shapiro Wilk test. All results were analyzed with a two-way ANOVA test, followed by a Bonferroni *post hoc* test, and data are presented as arithmetic means ± standard deviation. The level of significance was set at *P* < 0.05. The analysis was performed with GraphPad Prism 8.0.2 (San Diego, CA, USA).

## Results

### Changes in body mass and blood glucose level

The average baseline weight of female rats was 337.82 ± 3.80 g and that of male rats was 556.31 ± 4.66 g. The results are presented as percent change of body weight (final body weight vs baseline body weight).

**Figure 1:**
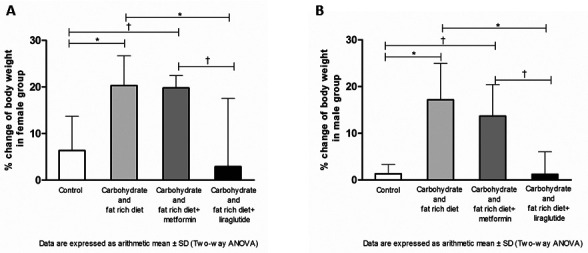
The percentage (%) of the body weight change at the end of the protocol compared with baseline values in female (**A**) and male (**B**) rats. Female groups (control N = 5, carbohydrate- and fat-rich diet [HSHFD] N = 4, HSHFD+metformin N = 4, HSHFD+liraglutide N = 5) and male groups (control N = 7, HSHFD N = 6, HSHFD+metformin N = 8, HSHFD+liraglutide N = 8). Data are presented as arithmetic mean ± standard deviation (SD) (two-way ANOVA *P* = 0.8852, F = 0.2153).

**Figure 2:**
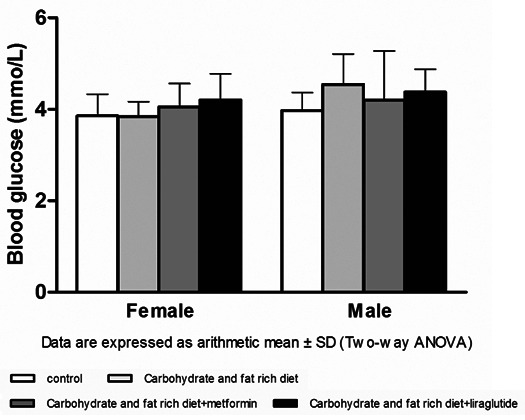
Blood glucose values (mmol/L) at the end of the protocol for all test groups of female and male rats. This part of the experiment included 28 female rats (control group N = 6, carbohydrate- and fat-rich diet [HSHFD] N = 8, HSHFD+metformin group N = 7, HSHFD+liraglutide group N = 7) and 29 male rats (control group N = 7, HSHFD group N = 6, HSHFD+metformin group N = 8, HSHFD+liraglutide group N = 8). Data are presented as arithmetic mean ± standard deviation (SD) (two-way ANOVA *P* = 0.5419, F = 0.7251)

Animals of both sexes in the HSHFD group (20.87% female group, *P* < 0.05, and 16.88% of the male group, *P* < 0.001) and HSHFD+metformin group (19.53% of the female group, *P* < 0.05, and 12.05% of the male group, *P* < 0.01) exhibited a significant increase in the percent change of body weight compared with the baseline (6.00% female group and 1.01% male group), which led to obesity development. Liraglutide treatment (2.23% female group and 5.16% male group) significantly reduced the percentage of body weight change in both sexes compared with the HSHFD group (20.87% of the female group, *P* < 0.01, and 16.88% of the male group, *P* < 0.001) and HSHFD+metformin group (19.53% of the female group, *P* < 0.01 and 12.05% of the male group, *P* < 0.01) (Figure 1). In neither of the sexes, significant differences in glucose concentration were found at the end of the protocol (Figure 2).

### mRNA gene expression in BBV (microcirculation) and aortas (macrocirculation)

The relative gene expression of *Gpx1* (*P* < 0.01) and *Gpx4* (*P* < 0.05) was significantly increased in control male aortas compared with control female aortas. *Cu/Zn Sod* gene expression was significantly increased (*P* < 0.001) in control male BBV compared with control female BBV. *MnSod* gene expression was significantly increased in HSHFD male BBV compared with HSHFD female BBV (*P* < 0.05). *Gpx1* gene expression was significantly decreased in control male BBV compared with control female BBV (*P* < 0.001) (Table 1). *Cu/Zn Sod* transcription was significantly increased in female aortas in the HSHFD+liraglutide group compared with the HSHFD and HSHFD+metformin groups (*P* < 0.05) and *Cat* gene expression compared with other groups (*P* < 0.001) (Table 2). *EC-Sod* (*P* < 0.05) and *Gpx1* (*P* < 0.05) gene expression in male HSHFD+metformin group aortas was significantly decreased compared with the control group. *Gpx4* gene expression in male HSHFD+metformin aortas was significantly decreased compared with the HSHFD+liraglutide group (*P* < 0.05). *Gpx1* gene expression in HSHFD and HSHFD+metformin male aortas was significantly decreased compared with the control group (*P* < 0.05) (Table 3)

**Table 1:** Antioxidant enzymes relative gene expression in female and male aortas and brain blood vessels (BBV). Results are presented as relative expression of gene normalized to hypoxanthine-guanine phosphoribosyltransferase as a reference gene and summarized as arithmetic mean ± standard deviation (two-way ANOVA)

Aortas	Cu/Zn Sod*		*MnSod^†^*		*EC-Sod^‡^*		*Gpx1^§^*		*Gpx4^‖^*		*Cat^⁋^*	
	female	male	female	male	female	male	female	male	female	male	female	male
Control	0.99 ± 0.64 (N = 4)	0.57 ± 0.62 (N = 5)	0.21 ± 0.41 (N = 5)	0.10 ± 0.09 (N = 5)	0.23 ± 0.29 (N = 5)	0.72 ± 0.80 (N = 4)	0.09 ± 0.05 (N = 5)	0.70 ± 0.52*** (N = 5)	0.19 ± 0.22 (N = 5)	0.61 ± 0.43*** (N = 6)	1.01 ± 0.82 (N = 5)	1.45 ± 0.51 (N = 5)
Carbohydrate- and fat-rich diet (HSHFD)	0.61 ± 0.34 (N = 6)	0.34 ± 0.24 (N = 6)	0.09 ± 0.08 (N = 7)	0.08 ± 0.05 (N = 5)	0.15 ± 0.08 (N = 6)	0.37 ± 0.27 (N = 5)	0.08 ± 0.04 (N = 7)	0.2 ± 0.25 (N = 5)	0.16 ± 0.12 (N = 7)	0.56 ± 0.36 (N = 6)	0.84 ± 0.51 (N = 5)	0.56 ± 0.37 (N = 4)
**BBV**	Cu/Zn Sod**		***MnSod***^††^		***EC-Sod****^‡‡^*		***Gpx1****^§§^*		***Gpx4****^‖‖^*		***Cat****^⁋⁋^*	
	female	male	female	male	female	male	female	male	female	male	female	male
Control	0.39 ± 0.13 (N = 6)	0.80 ± 0.06^†††^ (N = 5)	0.55 ± 0.20 (N = 6)	0.46 ± 0.60 (N = 5)	0.59 ± 0.34 (N = 6)	1.13 ± 0.95 (N = 5)	1.77 ± 0.71 (N = 6)	0.73 ± 0.12^†††^ (N = 5)	0.69 ± 0.47 (N = 6)	0.75 ± 0.28 (N = 5)	0.85 ± 0.27 (N = 6)	0.92 ± 0.11 (N = 5)
Carbohydrate- and fat-rich diet (HSHFD)	0.21 ± 0.15 (N = 6)	0.39 ± 0.13 (N = 5)	0.02 ± 0.003 (N = 4)	0.30 ± 0.16^‡‡‡^ (N = 6)	0.046 ± 0.04 (N = 8)	0.14 ± 0.09 (N = 5)	0.09 ± 0.09 (N = 6)	0.39 ± 0.30 (N = 6)	0.06 ± 0.03 (N = 8)	0.17 ± 0.05 (N = 5)	0.82 ± 0.37 (N = 4)	0.48 ± 0.47 (N = 5)

*Cu/Zn Sod (*P* = 0.7194, F = 0.1337).

†*MnSod* (*P* = 0.5165, F = 0.4379).

‡*EC-Sod* (*P* = 0.4627, F = 0.5662).

§*Gpx1* (*P* = 0.0285, F = 5.673).

‖‖*Gpx4* (*P* = 0.5229, F = 0.4230)

¶*Cat* (*P* = 0.4006, F = 0.7485).

**Cu/Zn Sod (*P* = 0.0311, F = 5.471).

††*MnSod* (*P* = 0.0151, F = 7.300).

‡‡*EC-Sod* (*P* = 0.2579, F = 1.356).

**§§***Gpx1* (*P* = 0.0008, F = 15.98).

**‖‖***Gpx4* (*P* = 0.7992, F = 0.06643).

¶¶*Cat* (*P* = 0.1835, F = 1.933).

***Male aortas control vs female aortas control (*Gpx1* *P* < 0.01; *Gpx4* *P* < 0.05), Bonferroni post hoc test, *P* < 0.05.

†††Male BBV control vs female BBV control (*Cu/Zn Sod* *P* < 0.001; *Gpx1* *P* < 0.05), Bonferroni post hoc test, *P* < 0.05.

‡‡‡Male BBV HSHFD vs female BBV HSHFD (*MnSod* *P* < 0.05), Bonferroni post hoc test, *P* < 0.05.

**Table 2:** Relative expression of superoxide dismutase isoforms (*Cu/Zn Sod*, *Mn Sod*, and *EC Sod*), glutathione peroxidase 1 and 4 (*Gpx1*, *Gpx4*), and catalase (*Cat*) genes in female aortas. Data are presented as arithmetic mean ± SD (two-way ANOVA)

Female aortas	Cu/Zn Sod*	*MnSod^†^*	*EC-Sod^‡^*	*Gpx1*^§^	*Gpx4^‖‖^*	*Cat^⁋^*
**Control**	0.99 ± 0.63 (N = 4)	0.09 ± 0.36 (N = 5)	0.23 ± 0.25 (N = 5)	0.09 ± 0.05 (N = 5)	0.19 ± 0.20 (N = 5)	1.01 ± 0.79^††^ (N = 5)
**Carbohydrate- and fat-rich diet (HSHFD)**	0.61 ± 0.33 (N = 6)	0.09 ± 0.08 (N = 7)	0.15 ± 0.08 (N = 6)	0.08 ± 0.04 (N = 7)	0.19 ± 0.11 (N = 7)	0.84 ± 0.50^††^ (N = 5)
**HSHFD+metformin**	0.55 ± 0.64**(N = 6)	0.07 ± 0.06 (N = 5)	0.15 ± 0.23 (N = 6)	0.08 ± 0.07 (N = 6)	0.04 ± 0.02 (N = 6)	1.12 ± 0.90^††^ (N = 7)
**HSHFD+liraglutide**	1.48 ± 0.33** (N = 4)	0.30 ± 0.20 (N = 4)	0.70 ± 0.55 (N = 4)	0.24 ± 0.18 (N = 4)	0.14 ± 0.10 (N = 5)	13.99 ± 2.92 (N = 4)

*Cu/Zn Sod (*P* = 0.1026, F = 2.22).

†*MnSod* (*P* = 0.7179, F = 0.4513).

‡*EC-Sod* (*P* = 0.2289, F = 1.512).

§*Gpx1* (*P* = 0.2512, F = 1.426).

*‖‖Gpx4* (*P* = 0.5166, F = 0.7716).

***¶****Cat* (*P* < 0.0001, F = 78.42).

**HSHFD+liraglutide vs HSHFD; HSHFD+metformin (*Cu/Zn Sod* *P* < 0.05), Bonferroni post hoc test, *P* < 0.05.

††HSHFD+liraglutide vs HSHFD; HSHFD+metformin; control (*Cat* *P* < 0.05), Bonferroni post hoc test, *P* < 0.05.

**Table 3:** Relative expression of superoxide dismutase isoforms (*Cu/Zn Sod*, *Mn Sod*, and *EC Sod*), glutathione peroxidase 1 and 4 (*Gpx1, Gpx4*), and catalase (*Cat*) genes in male aortas. Results are presented as relative expression of gene normalized to hypoxanthine-guanine phosphoribosyltransferase as a reference gene and summarized as arithmetic mean ± SD (two-way ANOVA)

Male aortas	Cu/Zn Sod*	*MnSod^†^*	*EC-Sod^‡^*	*Gpx1^§^*	*Gpx4^‖‖^*	*Cat^¶^*
**Control**	0.57 ± 0.62 (N = 5)	0.10 ± 0.09 (N = 5)	0.72 ± 0.80 (N = 4)	0.91 ± 0.65 (N = 5)	0.76 ± 0.53 (N = 6)	1.22 ± 0.67 (N = 5)
**Carbohydrate- and fat-rich diet (HSHFD)**	0.34 ± 0.23 (N = 6)	0.08 ± 0.04 (N = 5)	0.37 ± 0.27 (N = 5)	0.21 ± 0.24^††^ (N = 5)	0.56 ± 0.49 (N = 6)	0.56 ± 0.36 (N = 4)
**HSHFD+metformin**	0.69 ± 0.61 (N = 7)	0.10 ± 0.12 (N = 7)	0.08 ± 0.06** (N = 7)	0.33 ± 0.47^††^ (N = 7)	0.31 ± 0.25 (N = 8)	0.56 ± 0.48 (N = 7)
**HSHFD+liraglutide**	0.55 ± 0.37 (N = 7)	0.21 ± 0.24 (N = 6)	0.49 ± 0.57 (N = 5)	0.62 ± 0.78 (N = 5)	0.92 ± 0.84^‡‡^ (N = 6)	0.97 ± 0.93 (N = 5)

*Cu/Zn Sod (p=0.1026, F=2.22).

†*MnSod* (*P* = 0.7179, F = 0.4513).

‡*EC-Sod* (*P* = 0.2289, F = 1.512).

§*Gpx1* (*P* = 0.2512, F = 1.426).

*‖‖Gpx4* (*P* = 0.5166, F = 07716).

***¶****Cat* (*P* < 0.0001, F = 78.42).

**HSHFD+metformin vs control (*EC-Sod* *P* < 0.05), Bonferroni post hoc test, *P* < 0.05.

^††^HSHFD+metformin, HSHFD vs control (*Gpx1* *P* < 0.05), Bonferroni post hoc test, *P* < 0.05.

^‡‡^HSHFD+metformin vs HSHFD+liraglutide (*Gpx4* *P* < 0.05), Bonferroni post hoc test, *P* < 0.05.

In female BBV, *MnSod* gene expression (*P* < 0.001) was significantly decreased in all HSHFD groups (with or without treatment), and *EC-Sod* (*P* < 0.05) and *Gpx4* (*P* < 0.05) were decreased in the HSHFD and HSHFD+metformin groups compared with the control group. Metformin and liraglutide increased *Gpx1* gene expression (*P* < 0.05) compared with the HSHFD group. *Gpx4* gene expression in female BBV was significantly increased in the HSHFD+liraglutide group compared with the control (*P* < 0.05), HSHFD, and HSHFD+metformin groups (*P* < 0.001). *Cat* gene expression was significantly increased in the HSHFD+liraglutide group compared with the control group (*P* < 0.05) (Table 4).

**Table 4:** Relative expression of superoxide dismutase isoforms *Cu/Zn Sod*, *Mn Sod*, and *EC Sod*), glutathione peroxidase 1 and 4 (*Gpx1, Gpx4*), and catalase (*Cat*) genes in female brain blood vessels (BBV). Results are presented as relative expression of gene normalized to hypoxanthine-guanine phosphoribosyltransferase as a reference gene and summarized as arithmetic mean ± SD (two-way ANOVA)

Female BBV	Cu/Zn Sod*	*MnSod^†^*	*EC-Sod^‡^*	*Gpx1*^§^	*Gpx4^‖‖^*	*Cat^¶^*
**Control**	0.39 ± 0.12 (N = 6)	0.55 ± 0.19 (N = 6)	0.59 ± 0.33 (N = 6)	1.77 ± 0.70 (N = 6)	0.69 ± 0.46 (N = 6)	0.85 ± 0.26 (N = 6)
**Carbohydrate- and fat-rich diet (HSHFD)**	0.21 ± 0.15 (N = 6)	0.02 ± 0.003** (N = 4)	0.05 ± 0.04^††^ (N = 8)	0.09 ± 0.08** (N = 6)	0.06 ± 0.03^††^*^‖‖‖‖^* (N = 8)	0.82 ± 0.36 (N = 4)
**HSHFD+metformin**	0.33 ± 0.22 (N = 4)	0.07 ± 0.05** (N = 4)	0.04 ± 0.02^††^ (N = 6)	0.74 ± 0.62**^‡‡^ (N = 4)	0.07 ± 0.03^††^*^‖‖‖‖^* (N = 7)	0.92 ± 0.42 (N = 4)
**HSHFD+liraglutide**	0.49 ± 0.29 (N = 4)	0.13 ± 0.10** (N = 4)	0.11 ± 0.07 (N = 5)	0.91 ± 0.33**^‡‡^ (N = 4)	1.15 ± 0.73^§§^ (N = 5)	1.37 ± 0.56^¶¶^ (N = 5)

*Cu/Zn Sod (*P* = 0.207, F = 1.607).

†*MnSod* (*P* = 0.0011, F = 6.819).

‡*EC-Sod* (*P* = 0.494, F = 0.8142).

§*Gpx1* (*P* = 0.0023, F = 5.935).

*‖‖Gpx4* (*P* = 0.0002, F = 8.314).

***¶***Cat (*P* = 0.0459, F = 2.982).

**HSHFD; HSHFD+metformin; HSHFD+liraglutide vs control (*MnSod* *P* < 0.001), Bonferroni *post hoc* test, *P* < 0.05.

††HSHFD; HSHFD+metformin vs control (*EC-Sod* *P* < 0.05; Gpx4 *P* < 0.05), Bonferroni *post hoc* test, *P* < 0.05.

‡‡HSHFD+metformin; HSHFD+liraglutide vs HSHFD (*EC-Sod* *P* < 0.05), Bonferroni *post hoc* test, *P* < 0.05.

§§HSHFD+liraglutide vs control (*Gpx4* *P* < 0.05).

*‖‖‖‖*HSHFD+metformin; HSHFD vs HSHFD+liraglutide (*Gpx4* *P* < 0.001).

¶¶HSHFD+liraglutide vs control (*Cat* *P* < 0.05).

*Cu/Zn Sod* gene expression in male BBV was significantly decreased in the HSHFD group compared with other groups (*P* < 0.05). *MnSod* gene expression was significantly increased in the HSHFD+liraglutide group compared with the HSHFD and HSHFD+metformin groups (*P* < 0.01). Relative gene expression of *EC-Sod* and *Gpx4* was significantly decreased in all HDHFD groups compared with controls (*P* < 0.05) (Table 5).

**Table 5:** Relative expression of superoxide dismutase isoforms (*Cu/Zn Sod*, *Mn Sod,* and *EC Sod*), glutathione peroxidase 1 and 4 (*EC-Sod*, Gpx4), and catalase (*Cat*) genes in male brain blood vessels (BBV). Results are presented as relative expression of gene normalized to hypoxanthine-guanine phosphoribosyltransferase as a reference gene and summarized as arithmetic mean ± SD (two-way ANOVA)

Male BBV	Cu/Zn Sod*	*MnSod^†^*	*EC-Sod^‡^*	*Gpx1^§^*	*Gpx4^‖‖^*	*Cat^⁋^*
**Control**	0.78 ± 0.06** (N = 5)	0.70 ± 0.55 (N = 5)	1.13 ± 0.95 (N = 5)	0.73 ± 0.11 (N = 5)	0.75 ± 0.27 (N = 5)	0.92 ± 0.11 (N = 5)
**Carbohydrate- and fat-rich diet (HSHFD)**	0.55 ± 0.63 (N = 5)	0.30 ± 0.16^††^ (N = 6)	0.14 ± 0.09^‡‡^ (N = 5)	0.39 ± 0.30 (N = 6)	0.17 ± 0.05^‡‡^ (N = 5)	0.48 ± 0.47 (N = 5)
**HSHFD+metformin**	0.60 ± 0.06** (N = 5)	0.27 ± 0.68^††^ (N = 5)	0.27 ± 0.27^‡‡^ (N = 6)	0.50 ± 0.13 (N = 6)	0.21 ± 0.14^‡‡^ (N = 6)	0.51 ± 0.11 (N = 5)
**HSHFD+liraglutide**	0.62 ± 0.20** (N = 5)	0.60 ± 0.06 (N = 6)	0.36 ± 0.12^‡‡^ (N = 5)	0.62 ± 0.26 (N = 5)	0.20 ± 0.05^‡‡^ (N = 6)	0.54 ± 0.26 (N = 6)

*Cu/Zn Sod (*P* = 0.207, F = 1.607).

†*MnSod* (*P* = 0.0011, F = 6.819).

‡*EC-Sod* (*P* = 0.494, F = 0.8142).

§*Gpx1* (*P* = 0.0023, F = 5.935).

*‖‖Gpx4* (*P* = 0.0002, F = 8.314).

***¶****Cat* (*P* = 0.0459, F = 2.982).

**P < 0.05 HSHFD vs control; HSHFD+metformin; HSHFD+liraglutide (*Cu/Zn Sod* *P* < 0.05), Bonferroni post hoc test, *P* < 0.05.

††*P* < 0.05 HSHFD+liraglutide vs HSHFD; HSHFD+metformin (*MnSod* *P* < 0.01), Bonferroni *post hoc* test, *P* < 0.05.

‡‡*P* < 0.05 HSHFD; HSHFD+metformin; HSHFD+liraglutide vs control (*EC-Sod* *P* < 0.05, Gpx4 *P* < 0.05), Bonferroni *post hoc* test, *P* < 0.05.

### Serum levels of antioxidant enzymes activity

**Figure 3:**
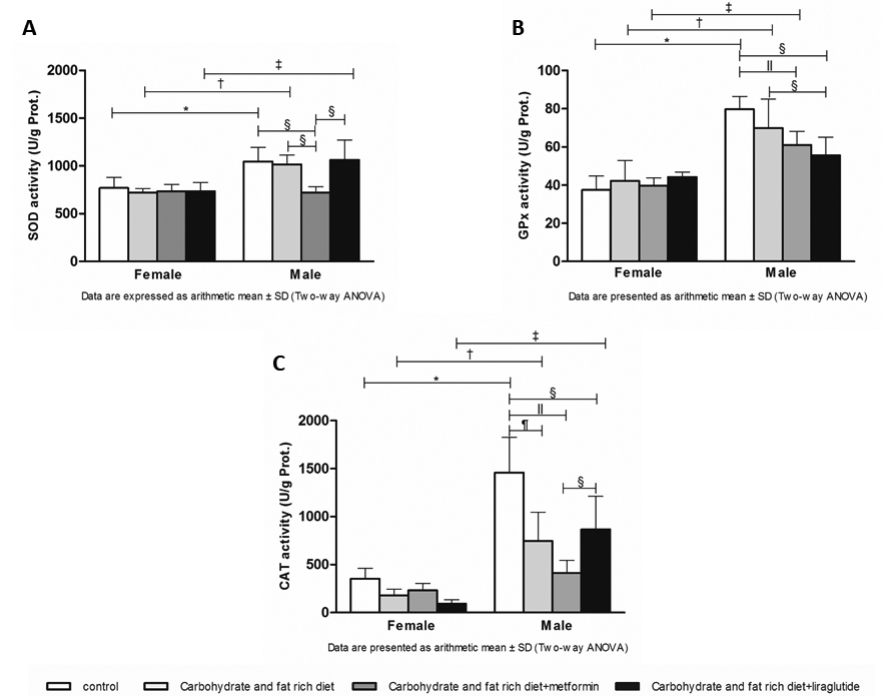
Comparison of serum antioxidative enzymes activities (SOD [**A**], GPx [**B**], and CAT [**C**]) between the sexes. The number of female samples for the measurement of serum SOD (control group N = 5, carbohydrate- and fat-rich diet [HSHFD] N = 7, HSHFD+metformin group N = 7, HSHFD+liraglutide group N = 4), GPx (control group N = 6, HSHFD N = 8, HSHFD+metformin group N = 7, HSHFD+liraglutide group N = 5), and CAT activity (control group N = 5, HSHFD N = 6, HSHFD+metformin group N = 6, HSHFD+liraglutide group N = 3). The number of male samples for the measurement of SOD (control group N = 6, HSHFD N = 6, HSHFD+metformin group N = 8, HSHFD+liraglutide group N = 8), GPx (control group N = 7, HSHFD N = 5, HSHFD+metformin group N = 8, HSHFD+liraglutide group N = 8), and CAT activity (control group N = 7, HSHFD N = 4, HSHFD+metformin group N = 6, HSHFD+liraglutide group N = 6). Data are presented as arithmetic mean ± standard deviation (SD) (two-way ANOVA: SOD P = 0.0013, F = 6.201; GPx P = 0.0004, F = 7.325; CAT P = 0.0003, F = 8.274).

Serum SOD, GPx, and CAT activity did not differ significantly among female groups. Within male groups, SOD activity was significantly decreased in the HSHFD+metformin group compared with other groups (*P* < 0.001). GPx activity in the HSHFD+liraglutide male group was significantly decreased compared with the control and HSHFD groups (*P* < 0.05) and in the HSHFD+metformin group compared with controls (*P* < 0.001). CAT activity in the HSHFD+liraglutide male group was significantly increased compared with the HSHFD+metformin male group (*P* < 0.01) and decreased compared with the control male group (*P* < 0.001). CAT activity in the HSHFD (*P* < 0.001) and HSHFD+metformin male group (*P* < 0.001) was significantly decreased compared with the control male group (Figure 3).

SOD and CAT activities were significantly increased in the male control (*P* < 0.01), HSHFD (*P* < 0.01), and HSHFD+liraglutide (*P* < 0.01) group compared with the corresponding female groups (Figure 3A and 3C). GPx1 activity in the male group was significantly increased in the control (*P* < 0.001), HSHFD group (*P* < 0.001), and HSHFD+metformin group (*P* < 0.001) compared with the corresponding female groups (Figure 3B).

### Oxidative stress and antioxidative capacity in serum samples

**Figure 4:**
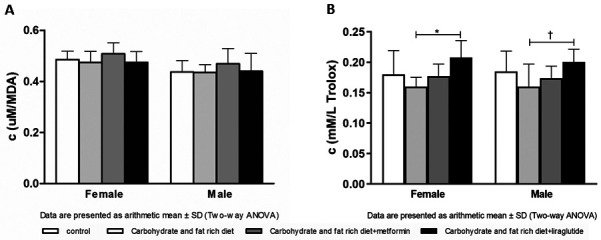
Indicators of oxidative stress. Comparison of thiobarbituric acid reactive substances (TBARS) values (**A**) and ferric reducing ability of plasma (FRAP) (**B**) between the sexes. The number of samples for TBARS per group: female groups (control N = 5, carbohydrate- and fat-rich diet [HSHFD] N = 7, HSHFD+metformin N = 7, HSHFD+liraglutide N = 5) and male groups (control N = 6, HSHFD N = 6, HSHFD+metformin N = 8, HSHFD+liraglutide N = 8) and for FRAP measurements in female groups (control N = 5, HSHFD N = 7, HSHFD+metformin N = 7, HSHFD+liraglutide N = 5) and male groups (control N = 6, HSHFD N = 6, HSHFD+metformin N = 7, HSHFD+liraglutide N = 8). Data are presented as arithmetic mean ± standard deviation (SD) (two-way ANOVA: TBARS *P* = 0.9880, F = 0.04305; FRAP *P* = 0.9493, F = 0.1175).

Oxidative stress (TBARS) level did not change significantly between groups or sexes (Figure 4A). Antioxidant capacity (FRA*P* values) was significantly increased in the HSHFD+liraglutide group (female *P* < 0.01; male *P* < 0.05) compared with the HSHFD group in both sexes. The level of antioxidant capacity in all studied groups did not significantly differ between the sexes (Figure 4B).

## Discussion

The main study findings of study performed in male and female rats are as follows: a) HSHFD diet increased body weight in both sexes; b) liraglutide treatment prevented the increase in body weight induced by HSHFD, while this effect was not observed with metformin treatment; c) liraglutide treatment significantly increased antioxidative capacity compared with the HSHFD group in both sexes; d) the activity of antioxidative enzymes was generally lower in females than in males; e) in male groups, HSFHD with or without antidiabetic therapy significantly lowered the activity of antioxidant enzymes compared with the control group; f) changes of antioxidative enzymes’ gene expression were more prominent in microvessels. All together, these results support the hypothesis that weight gain due to sugar- and fat-rich diet is crucial in developing oxidative stress due to inhibited antioxidative protective mechanisms.

GLP-1 receptors are found throughout the gastrointestinal tract, cardiomyocytes, vasculature, and the sinoatrial node (31-33). Newly developed agents acting through incretin hormones promote weight loss, in contrast to some oral antidiabetic agents (such as insulin secretagogues – sulfonylureas and meglitinides), thiazolidinediones, and insulin (34-36), which are associated with an increase in body weight (37). Our results are in concordance with these observations, showing that liraglutide prevented weight gain in animals fed with HSHFD compared with other obese groups of both sexes. These protective effects were not observed with metformin. Previous studies also found that patients with severe insulin resistance lost significantly more weight compared with insulin-sensitive patients (38). Additionally, our results showed that HSHFD increased glucose levels in neither of the sexes, which further confirms the metformin-related results. The similar blood glucose concentration among the groups observed in the present study is not in accordance with the results of a pre-diabetes rat model by Sheng et al (39). The authors showed that high-fat diet had a greater effect on glucose level and that high-sugar diet had a greater effect on blood triacylglycerol concentrations (39). The differences can be explained by a different fat and carbohydrate food content used in the two studies – while Sheng et al used food containing 20% fat and 20% of carbohydrates, the food in our study contained 56% carbohydrates and 12% of crude fat.

ROS, generated at sites of inflammation and damage, may cause cell damage and death. In vasculature, oxidative stress increases vascular endothelial permeability and promotes leukocyte adhesion (12). Our study did not find significantly increased serum TBARS levels, but it did observe an increased antioxidant capacity, showing a significant positive role of liraglutide in increasing the antioxidative status (19). Furthermore, liraglutide therapy and body weight reduction significantly increased the antioxidant capacity (FRAP values) compared with the HSHFD group in both sexes, which suggests an important antioxidant effect of liraglutide.

Although antioxidant enzyme activity in both sexes changed depending on the dietary protocol and therapy, it was lower in female groups. Enzyme activity was also modulated by liraglutide and metformin treatment. Individual studies examining sex differences and changes of antioxidant enzymes activity have shown that older male mice had a weakened link among three antioxidant enzymes (SOD, GPx, and CAT), regardless of lipid peroxidation concentration (40). However, in the liver and brain of older female mice, the cooperation between antioxidant enzymes was more coherent with increased lipid peroxidation concentration, which might explain why old females are better protected from oxidative stress than males (40). Our results suggest greater differences in enzyme activities among male groups, while antioxidative enzymes in female groups were not affected by dietary and pharmacological protocols. These findings suggest a more stable antioxidant status among females, which might explain their lower enzyme activity.

A limitation of our study was that the experimental design prevented us from performing a glucose tolerance test. Future studies could benefit from performing this test to establish a period of insulin resistance and to determine whether increased oxidative stress occurs before or after insulin resistance. Because of the strong impact of obesity and diabetes on the blood vessels reactivity (41-43) and endothelium-dependent responses (44,45), we examined the expression of antioxidant enzymes, which act as the first line of defense from high oxidative stress. The expression of antioxidant enzymes differed depending on the type of blood vessels and sex. Furthermore, it changed more significantly in the BBV of both sexes. A significant difference between the sexes in the gene expression of antioxidant enzymes was already observed between the control groups without treatment. HSHFD diet mostly affected MnSOD level only in the BBV. Higher expression of significant genes in the male than in the female group might explain higher enzyme activity in males.

In conclusion, we observed sex-related differences in oxidative stress level. Although we cannot determine which sex balances antioxidant status better based on gene expression and the level of antioxidant capacity alone, antioxidant enzymes activity in the female groups did not change significantly, indicating a more stable antioxidative status. The observed changes in oxidative status may be related to increased body weight, treatment preventing body weight gain, and oxidative stress increase. Liraglutide was more effective than metformin in regulating oxidative stress. The observed changes were more prominent in the microcirculation, supporting the observations of endothelial dysfunction in pre-diabetes and diabetes.

## References

[1] Lastra G, Manrique CM, Hayden MR (2006). The role of beta-cell dysfunction in the cardiometabolic syndrome.. J Cardiometab Syndr.

[2] Lozano I, Van der Werf R, Bietiger W, Seyfritz E, Peronet C, Pinget M (2016). High-fructose and high-fat diet-induced disorders in rats: impact on diabetes risk, hepatic and vascular complications.. Nutr Metab (Lond).

[3] Crescenzo R, Bianco F, Mazzoli A, Giacco A, Cancelliere R, di Fabio G (2015). Fat Quality influences the obesogenic effect of high fat diets.. Nutrients.

[4] Esposito K, Ciotola M, Giugliano D (2006). Oxidative stress in the metabolic syndrome.. J Endocrinol Invest.

[5] Roberts CK, Sindhu KK (2009). Oxidative stress and metabolic syndrome.. Life Sci.

[6] Sies H, Stahl W, Sevanian A (2005). Nutritional, dietary and postprandial oxidative stress.. J Nutr.

[7] Dandona P, Ghanim H, Chaudhur A, Dhindsa S, Kim SS (2010). Macronutrient intake induces oxidative and inflammatory stress: Potential relevance to atherosclerosis and insulin resistance.. Exp Mol Med.

[8] Serra D, Mera P, Malandrino MI, Mir JF, Herrero L (2013). Mitochondrial fatty acid oxidation in obesity.. Antioxid Redox Signal.

[9] Savini I, Catani MV, Evangelista D, Gasperi V, Avigliano L (2013). Obesity-associated oxidative stress: strategies finalized to improve redox state.. Int J Mol Sci.

[10] Ozata M, Mergen M, Oktenli C, Aydin A, Sanisoglu SY, Bolu E (2002). Increased oxidative stress and hypozincemia in male obesity.. Clin Biochem.

[11] Coutinho T, Goel K, Corrêa de Sá D, Carter RE, Hodge D, Kragelund C (2013). Combining body mass index with measures of central obesity in the assessment of mortality in subjects with coronary disease: Role of “normal weight central obesity”.. J Am Coll Cardiol.

[12] Hadi H, Carr C, Suwaidi J (2005). Endothelial dysfunction: Cardiovascular risk factors, therapy, and outcome.. Vasc Health Risk Manag.

[13] Couillard C, Ruel G, Archer WR, Pomerleau S, Bergeron J, Couture P (2005). Circulating levels of oxidative stress markers and endotelial adhesión molecules in men with abdominal obesity.. J Clin Endocrinol Metab.

[14] Paneni F, Costantino S, Cosentino F (2014). Insulin resistance, diabetes, and cardiovascular risk.. Curr Atheroscler Rep.

[15] Dandona P, Aljada A, Chaudhuri A, Mohanty P, Garg R (2005). Metabolic syndrome: A comprehensive perspective based on interactions between obesity, diabetes, and inflammation.. Circulation.

[16] Wook K, Egan MJ (2008). The role of incretins in glucose homeostasis and diabetes treatment.. Pharmacol Rev.

[17] Okada K, Kotani K, Yagyu H, Ando A, Osuga J, Ishibashi S (2014). Effects of treatment with liraglutide on oxidative stress and cardiac natriuretic peptide levels in patients with type 2 diabetes mellitus.. Endocrine.

[18] Oh YS, Jun HS (2017). Effects of glucagon-like peptide-1 on oxidative stress and nrf2 signaling.. Int J Mol Sci.

[19] Lotfy M, Singh J, Rashed H, Tariq S, Zilahi E, Adeghate E (2014). Mechanism of the beneficial and protective effects of exenatide in diabetic rats.. J Endocrinol.

[20] Yong OK, Detlef S (2012). When GLP-1 hits the liver: a novel approach for insulin resistance and NASH.. Am J Physiol.

[21] Almutairi M, Batran RA, Ussher JR (2019). Glucagon-like peptide-1 receptor action in the vasculature.. Peptides.

[22] Martin-Montalvo A, Mercken EM, Mitchell SJ, Palacios HH, Mote PL, Scheibye-Knudsen M (2013). Metformin improves healthspan and lifespan in mice.. Nat Commun.

[23] Dehkordi AH, Abbaszadeh A, Mir S, Hasanvand A (2019). Metformin and its anti-inflammatory and anti-oxidative effects; new concepts.. J Renal Inj Prev.

[24] Han J, Li Y, Liu X, Zhou T, Sun H, Edwards P (2018). Metformin suppresses retinal angiogenesis and inflammation in vitro and in vivo.. PLoS One.

[25] Cosic A, Jukic I, Stupin A, Mihalj M, Mihaljevic Z, Novak S (2016). Attenuated flow-induced dilatation of middle cerebral arteries is related to increased vascular oxidative stress in rats on a short-term high salt diet.. J Physiol Heart Cir Physiol.

[26] Matic A, Jukic I, Stupin A, Baric L, Mihaljevic Z, Unfirer S (2018). High salt intake shifts the mechanisms of flow- induced dilation in the middle cerebral arteries of Sprague-Dawley rats.. Am J Physiol.

[27] Mihaljević Z, Matić A, Stupin A, Barić L, Jukić I, Drenjančević I (2018). Acute hyperbaric oxygenation, contrary to intermittent hyperbaric oxygenation, adversely affects vasorelaxation in healthy Sprague- Dawley rats due to increased oxidative stress.. Oxid Med Cell Longev.

[28] Vuković R, Blažetić S, Oršolić I, Heffer M, Vari SG, Gajdoš M (2014). Impact of ovariectomy, high fat diet, and lifestyle modifications on oxidative/antioxidative status in the rat liver.. Croat Med J.

[29] Novak S, Drenjancevic I, Vukovic R, Kellermayer Z, Cosic A, Tolusic Levak M (2016). Anti-inflammatory effects of hyperbaric oxygenation during DSS-induced colitis in BALB/c mice include changes in gene transcription of HIF-1α, proinflammatory cytokines, and antioxidative enzymes.. Mediators Inflamm.

[30] Barić L, Drenjančević I, Mihalj M, Matić A, Stupin M, Kolar L (2020). Enhanced antioxidative defense by vitamins C and E consumption prevents 7-day high-salt diet-induced microvascular endothelial function impairment in young healthy individuals.. J Clin Med.

[31] Wei Y, Mojsov S (1995). Tissue-specific transcription of the human receptor for glucagon-like peptide-I: brain, heart and pancreatic forms have the same deduced amino acid sequences.. FEBS Lett.

[32] Pyke C, Heller RS, Kirk RK, Ørskov C, Reedtz-Runge S, Kaastrup P (2014). GLP-1 receptor localization in monkey and human tissue: novel distribution revealed with extensively validated monoclonal antibody.. Endocrinology.

[33] Richards P, Parker HE, Adriaenssens AE, Hodgson JM, Cork SC, Trapp S (2014). Identification and characterization of GLP-1 receptor-expressing cells using a new transgenic mouse model.. Diabetes.

[34] Scheen AJ (2003). Current management strategies for coexisting diabetes mellitus and obesity.. Drugs.

[35] Todd JF, Bloom SR (2007). Incretins and other peptides in the treatment of diabetes.. Diabet Med.

[36] Hermann LS, Kalen J, Katzman P, Lager I, Nilsson A, Norrhamn O (2001). Long-term glycaemic improvement after addition of metformin to insulin in insulin-treated obese type 2 diabetes patients.. Diabetes Obes Metab.

[37] Kim SW (2010). Triple Combination Therapy Using Metformin, Thiazolidinedione, and a GLP-1 analog or DPP-IV inhibitor in patients with type 2 diabetes mellitus.. Korean Diabetes J.

[38] Seifarth C, Schehler B, Schneider HJ (2013). Effectiveness of metformin on weight loss in non-diabetic individuals with obesity.. Exp Clin Endocrinol Diabetes.

[39] Liu Y, Wang Z, Xiang X, Zhang X, Yang Y (2014). Analysis of the effect of high glucose and high fat diet on the manufacturing of the experimental pre-diabetic rats model.. Wei Sheng Yan Jiu..

[40] Sobočanec S, Balog T, Kušić B, Šverko V, Šarić A, Marotti T (•••). Differential response to lipid peroxidation in male and female mice with age: Correlation of antioxidant enzymes matters. Biogerontology.

[41] Kibel A, Novak S, Ćosić A, Mihaljević Z, Falck JR, Drenjančević I (2015). Hyperbaric oxygenation modulates vascular reactivity to angiotensin-(1-7) in diabetic rats - potential role of epoxyeicosatrienoic acids.. Diab Vasc Dis Res.

[42] Manojlovic D, Stupin A, Mihaljevic Z, Matic A, Lenasi H, Drenjancevic I (2019). Hyperbaric oxygenation affects acetylcholine-induced relaxation in female diabetic rats.. Undersea Hyperb Med.

[43] Unfirer S, Mihalj M, Novak S, Kibel A, Čavka A, Mihaljević Z (2016). Hyperbaric oxygenation affects the mechanisms of acetylcholine-induced relaxation in diabetic rats.. Undersea Hyperb Med.

[44] Grizelj I, Čavka A, Bian JT, Szczurek M, Robinson A, Shinde S (2015). Reduced flow-and acetylcholine-induced dilations in visceral compared to subcutaneous adipose arterioles in human morbid obesity.. Microcirculation.

[45] Didion SP, Lynch CM, Baumbach GL, Faraci FM (2005). Impaired endothelium-dependent responses and enhanced influence of Rho-kinase in cerebral arterioles in type II diabetes.. Stroke.

